# Antimicrobial Susceptibility of Bacteria That Cause Bovine Respiratory Disease Complex in Alberta, Canada

**DOI:** 10.3389/fvets.2017.00207

**Published:** 2017-12-04

**Authors:** R. Michele Anholt, Cassidy Klima, Nick Allan, Heather Matheson-Bird, Crystal Schatz, Praseeda Ajitkumar, Simon JG Otto, Delores Peters, Karin Schmid, Merle Olson, Tim McAllister, Brenda Ralston

**Affiliations:** ^1^POV Inc., Airdrie, AB, Canada; ^2^Agriculture and Agri-Food Canada, Lethbridge, AB, Canada; ^3^Chinook Contract Research Inc., Airdrie, AB, Canada; ^4^Bow Valley Research Inc., Calgary, AB, Canada; ^5^Institute for Applied Poultry Technologies, Airdrie, AB, Canada; ^6^Alberta Ministry of Agriculture and Forestry, Airdrie, AB, Canada; ^7^School of Public Health, University of Alberta, Edmonton, AB, Canada; ^8^Alberta Beef Producers, Calgary, AB, Canada; ^9^Alberta Veterinary Laboratories Ltd, Calgary, AB, Canada

**Keywords:** antimicrobial resistance, bovine respiratory disease complex, *Mannheimia haemolytica*, *Mycoplasma bovis*, *Pasteurella multocida*, *Haemophilus somnus*, *Trueperella pyogenes*

## Abstract

Bovine respiratory disease (BRD) is the most important illness of feedlot cattle. Disease management targets the associated bacterial pathogens, *Mannheimia haemolytica, Mycoplasma bovis, Pasteurella multocida, Histophilus somni*, and *Trueperella pyogenes*. We conducted a cross-sectional study to measure the frequencies of antimicrobial-resistant BRD pathogens using a collaborative network of veterinarians, industry, government, and a diagnostic laboratory. Seven private veterinary practices in southern Alberta collected samples from both living and dead BRD-affected animals at commercial feedlots. Susceptibility testing of 745 isolates showed that 100% of the *M. haemolytica, M. bovis, P. multocida*, and *T. pyogenes* isolates and 66.7% of the *H. somni* isolates were resistant to at least one antimicrobial class. Resistance to macrolide antimicrobials (90.2% of all isolates) was notable for their importance to beef production and human medicine. Multidrug resistance (MDR) was high in all target pathogens with 47.2% of the isolates resistant to four or five antimicrobial classes and 24.0% resistance to six to nine classes. We compared the MDR profiles of isolates from two feedlots serviced by different veterinary practices. Differences in the average number of resistant classes were found for *M. haemolytica* (*p* < 0.001) and *P. multocida* (*p* = 0.002). Compared to previous studies, this study suggests an increasing trend of resistance in BRD pathogens against the antimicrobials used to manage the disease in Alberta. For the veterinary clinician, the results emphasize the importance of ongoing susceptibility testing of BRD pathogens to inform treatment protocols. Surveillance studies that collect additional epidemiological information and manage sampling bias will be necessary to develop strategies to limit the spread of resistance.

## Introduction

Bovine respiratory disease (BRD) in newly received calves continues to be the most predominant health issue for North American beef production, with incidences that range from 5 to 44% and estimated costs to producers at $13.90 per animal (1). *Mannheimia haemolytica, Mycoplasma bovis, Pasteurella multocida, Histophilus somni*, and *Trueperella pyogenes* are all opportunistic bacterial agents that can be associated with BRD: the roles of each in BRD infections have been extensively reviewed elsewhere (2, 3). Microbiological testing adds to production costs and time-to-results limits the feasibility of using these tests to inform real-time treatment decisions. Consequently, the empirical use of broad-spectrum antimicrobials is currently considered essential for the prevention and treatment of BRD (4).

There is evidence of declining efficacy of the antimicrobials commonly used to manage these pathogens (5–7). Resistance to multiple antimicrobial classes has been associated with large, mobile genetic elements in *M. haemolytica* and *P. multocida* (8–10). Poor response to antimicrobial therapy threatens livestock health and welfare, may lead to increased antimicrobial use (AMU), increases production costs, and potentially contributes to the dissemination of antimicrobial-resistant genes to other bacteria in cattle and possibly the environment (11, 12).

Knowledge of regional antimicrobial sensitivity patterns can help veterinarians design effective treatment protocols, inform management strategies to support responsible antimicrobial stewardship, reduce costs of production, and improve animal health and welfare. The objectives of this study were to (i) investigate the feasibility of a collaborative network of private practice veterinarians, industry representatives, government agencies, and a diagnostic laboratory for monitoring antimicrobial resistance (AMR) in beef cattle and (ii) conduct a cross-sectional study to describe the pathogens isolated in clinical BRD cases and the frequency of AMR in the isolated pathogens.

## Materials and Methods

### Target Population and Sample Collection

Sixty commercial feedlots located in southern Alberta, managed by seven private veterinary practices, and ranging in capacity from 2,000 to 25,000 head, participated in the study. An estimation of the sample size needed to assess AMR to BRD pathogens was calculated. The assumptions were: (i) significance level, 0.05, (ii) *a priori* estimate of the proportion AMR positive, conservatively = 0.05, (iii) precision = 0.05. We estimated a 60% recovery of a targeted BRD pathogen from the collected samples. The estimated number of cattle to be sampled was 642. Sampling occurred between September 2014 and March 2015 and included all types, ages, and sexes of cattle.

Veterinarians and feedlot managers who participated in this study did so voluntarily with the assurance that we would respect the anonymity and confidentiality of their data. Most of the cattle sampled in this study had died as a result of disease. Live animal sampling was within the veterinary scope of practice for commercial beef production in Canada.

Samples were collected from both morbid cattle and those that had succumbed to BRD. Morbid cattle were sampled if pulled for treatment and diagnosed chute-side with BRD based on elevated rectal temperature (>40°C) and clinical signs consistent with the disease. Guarded, deep nasopharyngeal swabs (Jorgensen Laboratories, Inc., Loveland, CO, USA) were employed for live animal sampling. Swabs were stored in Amies^®^ bacterial transport medium (Starplex Scientific, Inc., Etobicoke, ON, USA) at 4°C until delivered to the diagnostic laboratory within 3 days of sampling. Samples were frozen and stored at −20°C until processing if delivery time was projected to exceed 72 h. Mortalities were sampled based on gross pathological evidence of infectious pneumonia at postmortem. Samples collected at postmortem included: lung tissue; nasal, tracheal, and laryngeal swabs; pleural fluid; heart or pericardium; joint fluid; peritoneal fluid and tissue; and abscesses. These were collected aseptically avoiding contamination by environmental bacteria and stored in sterile containers without media at 4°C until delivered to the diagnostic laboratory within 3 days of sampling from participating practices. Descriptive and clinical information regarding the sampled animal were also collected if possible (Table 1). All samples were processed at the Institute of Applied Poultry Technologies in Airdrie, AB, Canada.

**Table 1:** Variables collected for animals entered in the study.

Descriptor
Animal identification number
Ear tag number
Veterinary practice code
Farm
Region
Alive? (True or false)
Animal type (fall calves, winter calves, yearlings, adults)
Number of days on feed
Field diagnosis/diagnoses
Treatment on arrival
Additional treatment(s)

### Sample Processing

Swab samples were inoculated directly onto Tryptic Soy Agar containing 5% blood (TSA-B) (VWR, Mississauga, ON, Canada) and incubated 16–72 h at 37°C for isolation of *M. haemolytica, P. multocida*, and *T. pyogenes*. For the isolation of *H. somni*, swabs were inoculated directly onto Chocolate Agar (VWR, Mississauga, ON, Canada) and incubated for 16–72 h at 37°C with 5% CO_2_. For isolation of *M. bovis*, swabs were inoculated directly on either heart infusion Agar (Becton Dickenson, Sparks, MD, USA) containing 500 µg/mL ampicillin or modified Eaton’s Agar (13) containing 500 µg/mL ampicillin and incubated for 120 h at 37°C with 5% CO_2_.

Tissue samples were manually homogenized in 10 mL of brain heart infusion (BHI) broth for approximately 1 min or until even consistency was achieved. Both the homogenized tissue suspensions and raw fluid samples were serially diluted 1:10 in BHI broth. For isolation of *M. haemolytica, P. multocida*, and *T. pyogenes*, 100 µL of the 10^−1^ and 10^−2^ dilutions were each inoculated onto TSA-B, and on Chocolate Agar for the isolation of *H. somni* with the culture conditions as specified above. For isolation of *M. bovis*, the diluted, homogenized tissue suspensions, or joint fluid samples were each filtered through a coarse, large pore filter and then through a 0.45 µm filter syringe. A 100 µL aliquot of the resulting filtrate was added to 10 mL of sterile heart infusion (HI) broth containing 500 µg/mL ampicillin. Subsequently, a 100 µL of the resulting diluted, filtered sample was inoculated directly on either HI Agar containing 500 µg/mL ampicillin, or modified Eaton’s Agar containing 500 µg/mL ampicillin, and incubated for 120 h at 37°C with 5% CO_2_.

### Species Confirmation

Isolates displaying appropriate morphologies for *Mycoplasma, M. haemolytica, P. multocida*, and *H. somni* were species confirmed using Matrix Assisted Laser Desorption/Ionization-Time of Flight (Maldi-Tof) Mass Spectroscopy (microflex™ LT/SH Maldi-TOF, Bruker Corp., Milton, ON, Canada) according to manufacturer’s specifications. Resulting spectra were analyzed using Maldi BioTyper^®^ software (Bruker Daltonik GmbH, Leipzig, Germany). Samples stored for later analysis were preserved using Microbank™ cryopreservation carriers (Pro-Lab Diagnostics, Richmond Hill, ON, Canada) following manufacturer guidelines.

### Antimicrobial Susceptibility Testing (AST)

Antimicrobial susceptibility testing was performed on all isolates using broth microdilution and a commercially available bovine/porcine panel (Sensititre; Trek Diagnostic Systems, Cleveland, OH, USA) and standardized breakpoints. Briefly, all isolates were suspended in 0.9% saline to a McFarland standard of 0.5 with 10 µL of the resulting suspension used to inoculate 11 mL of Mueller-Hinton Broth with TES containing lysed horse blood (Trek Diagnostic Systems, Cleveland, OH, USA). This final suspension was used to inoculate the Sensititre plates per the manufacturer’s instructions. For *M. haemolytica, P. multocida*, and *T. pyogenes*, plates were sealed and incubated at 34−37°C aerobically for 18−24 h. *H. somni and M. bovis* plates were sealed using perforated film and incubated with 5% CO_2_ at 34−37°C for 24−96 h. Minimum inhibitory concentrations (MIC50) were assigned by eye as outlined in the Clinical and Laboratory Standards Institute (14). Where CLSI breakpoints were unavailable, susceptibility was estimated from available CLSI recommendations for human pathogen/antimicrobial combinations (15).

### Analysis

The proportion of samples positive for *M. haemolytica, P. multocida, T. pyogenes, H. somni*, and *M. bovis* (target organisms) and the distributions of MIC’s for each pathogen/antimicrobial combination were determined. Quantitative levels of susceptibility were measured using the 50th percentiles for MIC50. Multidrug resistance (MDR) isolates were defined as resistance observed to multiple drug classes with extreme MDR (XDR) defined as any isolate resistant to six or more classes. Lacking a cell wall, *M. bovis* is inherently resistant to the β-lactams so resistance to this class was not included.

Two feedlots (Feedlot A and Feedlot B) serviced by different veterinary practices provided a substantial number of samples (15.2 and 33.2% of total samples, respectively) with which we could compare MDR profiles. For each of the pathogens, two sample Wilcoxon rank-sum test was used to compare the MDR at Feedlot A to Feedlot B.

## Results

### Animals and Samples Collected

**Figure 1:**
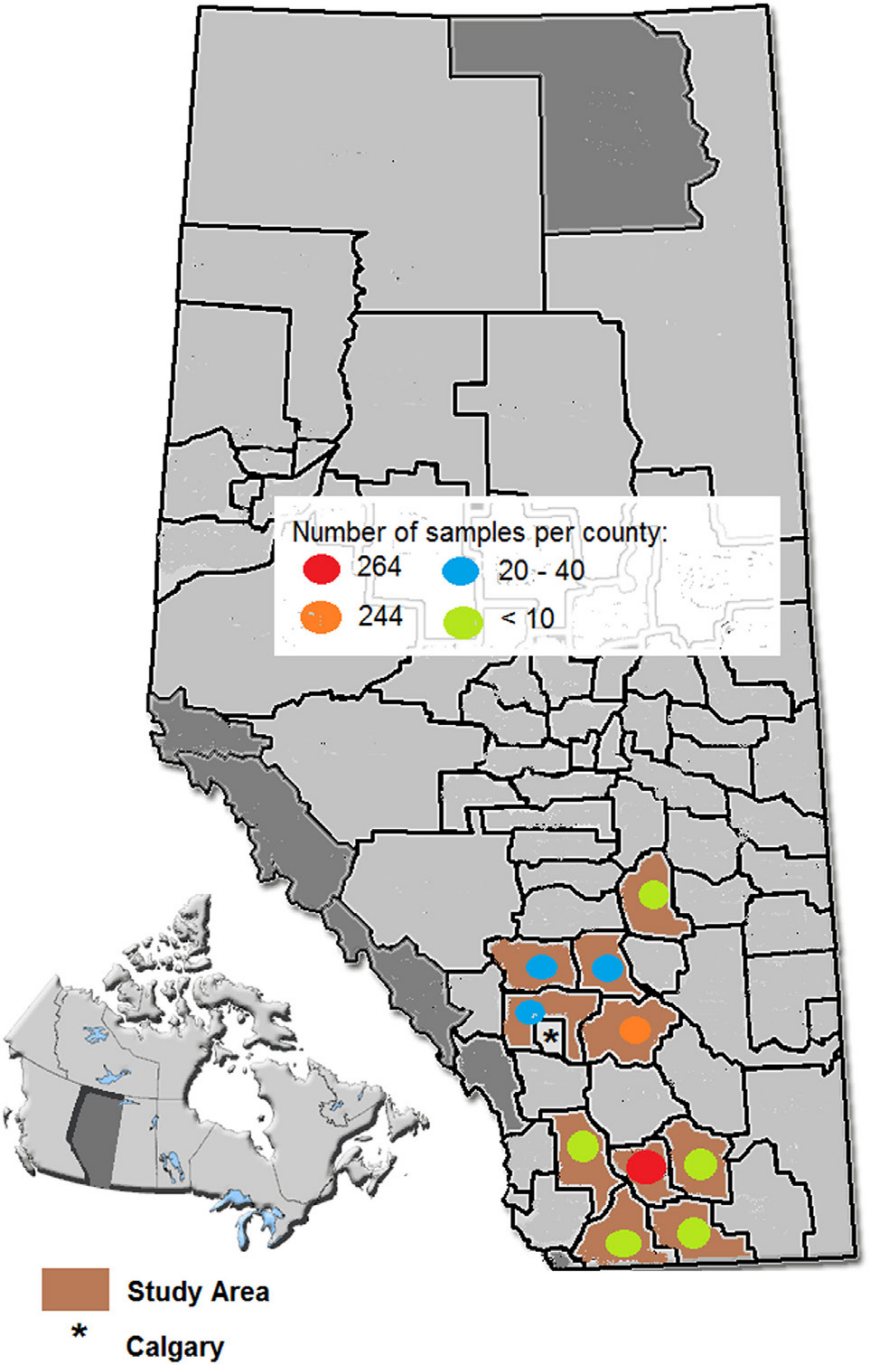
Map of the study area.

The seven participating practices submitted samples from 60 feedlots located in 10 municipal counties in southern Alberta (Figure 1). Samples for microbiological analysis (*n* = 740) were collected from 618 animals (528 mortalities and 90 morbid cattle), slightly fewer than the calculated sample size. Time constraints prevented further sampling. Animal age was recorded for 76.3% of the cases and where provided, 90.5% of animals were calves (less than one year of age), 8.1% were yearlings, and 1.4% were adults (2 years and older). The number of days on feed was provided for 86.6% of the animals, with 56.7% of these collected between 0 and 30 days on feed, 21.4% between 31 and 60 days on feed, and 22% greater than 60 days on feed. A field diagnosis was provided for 99% of the animals with target-positive samples; 94.5% were described as pneumonia and/or pleuritis. Arthritis and pericarditis/myocarditis, usually as comorbidities, were identified in 5.7 and 3.6% of the cattle, respectively.

### Microbiological Results

One or more of the targeted pathogens (*n* = 775) were isolated from 457 cattle and AMR susceptibility testing was completed on 745 target isolates from 441 animals (Table 2). Overall, 73.9% of the animals sampled resulted in recovery of a targeted pathogen. Percent recovery of the target organisms varied by sample and organism (Table 2). Lung samples returned primarily *M. haemolytica* and M. *bovis* isolates with 104 samples yielding both organisms. Of these samples, 78 (75%) of the field diagnoses were fibrinous pneumonia and the remaining samples were chronic pneumonia, bronchopneumonia, or not specified (e.g., BRD). Swab samples yielded proportionally larger populations of *P. multocida* than that observed in the lower respiratory tract. *Trueperella pyogenes* was recovered most frequently from deep nasal swab as well as from joints. Both *M. bovis* and *H. somni* were most readily isolated from heart/pericardium tissues.

**Table 2:** Samples collected, isolates recovered, and isolates used in susceptibility analysis (*n* = 618 animals).

Sample	Isolate	No. isolates	% recovery	No. isolates with antimicrobial resistance results
Lung (*n* = 480)	*Mannheimia haemolytica*	213	44.4	208
	*Mycoplasma bovis*	198	41.3	194
	*Pasteurella multocida*	86	17.9	85
	*Histophilus somni*	64	13.3	63
	*Trueperella pyogenes*	69	14.4	69

Pleural fluid (*n* = 20)	*M. haemolytica*	7	35	7
	*M. bovis*	1	5	1
	*P. multocida*	0	0	0
	*H. somni*	1	5	1
	*T. pyogenes*	0	0	0

Nasal swab (*n* = 75)	*M. haemolytica*	14	18.7	6
	*M. bovis*	8	10.7	7
	*P. multocida*	15	20	15
	*H. somni*	2	2.7	2
	*T. pyogenes*	0	0	0

Deep nasal swab (*n* = 71)	*M. haemolytica*	15	21.1	10
	*M. bovis*	16	22.5	16
	*P. multocida*	15	21.1	15
	*H. somni*	5	7	3
	*T. pyogenes*	15	21.1	15

Laryngeal/tracheal swab (*n* = 4)	*M. haemolytica*	0	0	0
	*M. bovis*	1	25	1
	*P. multocida*	1	25	1
	*H. somni*	0	0	0
	*T. pyogenes*	2	50	2

Heart/pericardium (*n* = 18)	*M. haemolytica*	1	5.5	1
	*M. bovis*	4	22.2	2
	*P. multocida*	0	0	0
	*H. somni*	6	33.3	5
	*T. pyogenes*	1	5.4	1

Peritoneum (*n* = 1)	*M. haemolytica*	0	0	0
	*M. bovis*	1	100	1
	*P. multocida*	0	0	0
	*H. somni*	0	0	0
	*T. pyogenes*	0	0	0

Joint fluid (*n* = 70)	*M. haemolytica*	1	1.4	1
	*M. bovis*	4	5.7	3
	*P. multocida*	0	0	1
	*H. somni*	1	1.4	0
	*T. pyogenes*	7	10	7

Abscess (*n* = 1)	*M. haemolytica*	0	0	0
	*M. bovis*	1	100	1
	*P. multocida*	1	100	0
	*H. somni*	1	100	1
	*T. pyogenes*	0	0	0

Treatment data on entry to the feedlot was provided for 46.6% of the submissions and of these, to 95.5% of the animals were administered a macrolide antimicrobial. Information about in-feed antimicrobials was not collected but feedlots in Western Canada routinely add tetracyclines to the feed during the early (approximately 10–20 days) feeding period for BRD prevention, and either tylosin or tetracyclines throughout the feeding period for liver abscess control (C. Dorin, personal communication, November 2015).

### Antimicrobial Resistance

There was AMU information provided for 444 treatments from 235 (38%) animals. The number of antimicrobial treatments per animal, including entry, ranged from 1 to 5. The average number of antimicrobial classes represented in the treatments was 1.86 (SD, 0.99). In the cattle selected for treatment, florfenicol was used in 35.4% of cases, enrofloxacin in 27.5%, and ceftiofur in 14%. Macrolides (8.8%), tetracyclines (7.4%), and trimethoprim sulfas (7.0%) were used in the remaining treated cattle.

The distributions of the MIC’s for the five target organisms are provided in Tables 3–7. Based on the breakpoints used, there was a low frequency of resistance to the Category I antimicrobials (agents of very high importance to human health—ceftiofur, enrofloxacin, and danofloxacin). However, a high frequency of resistance to Category II antimicrobials (high importance to human health—neomycin, tylosin, tulathromycin, tilmicosin, and clindamycin), was observed especially among *M. haemolytica, M. bovis*, and *P. multocida* isolates.

**Table 3:** Distribution of minimum inhibitory concentrations among *Mannheimia haemolytica* isolates (*n* = 219 animals, 233 samples: lung samples = 208, nasal swabs = 16, pleural fluid = 7, heart = 1, joint fluid = 1).

					Distribution (%) of MICs (μg/mL)									
Class	Category^a^	Antimicrobial	MIC 50	%R^b^	≤0.12	0.25	0.5	1	2	4	8	16	32	64
Aminoglycoside	II	GEN	4	3.4^c^				4.3	38.6	50.6	3.0	3.4		
	II	NEO	8	49.4^c^						13.7	36.9	1.3	48.1	
	III	SPE	32	4.3							1.7	20.2	73.0	64 = 0.9 >64 = 4.3
Fluorquinolone	I	ENRO	0.12	3.0	94	1.3	1.3	0.4	3.0					
	I	DANO	0.12	3.9^c^	91	4.3	0.9	3.9						
Macrolide	II	TYLT	32	99.1^c^						0.4	0.4	4.3	94.8	
	II	TUL	16	37.8				4.3	6.9	14.6	17.6	14.2	4.7	37.8
	II	TIL	16	44.2						34.8	8.6	12.4	5.6	38.6
B lactams	I	XNL	0.25	0.9		96.6	0.4	1.7	0.4		0.9			
	II	PEN	0.12	7.2	51.9	36.5	4.3	1.7	0.4	0.4	4.7			
	II	AMP	0.25	5.1^c^		92.3	1.3	1.3	0.9	0.4	0.4	3.4		
Lincosamides	II	CLIN	8	77.7^c^		0.9		0.9	2.1	18.5	57.5	20.2		
Phenicol	III	FFN	1	4.3		1.7	39.9	51.1	2.1	0.9	4.3			
Tetracycline	III	OXY	8	53.6			20.6	20.6	4.3	0.9	53.6			
	III	CTET	2	11.2			8.2	30.0	25.8	24.9	11.2			
Pleuromutilin	III	TIA	16	19.7^c^				0.4	1.7	3.9	9.4	64.8	19.7	

*Trimethoprim sulpha^c^ (Category II): <2/38 = 98.3%, >2/38 = 1.7%*.

*Sulphahdimethoxine^c^ (Category III): <256 = 44.6%, >256 = 55.4%*.

*GEN, gentamycin/NEO, neomycin/SPE, spectinomycin; DAN, danofloxacin/ENRO, enrofloxacin; TYLT, tylosin/TUL, tulathromycin/TIL, tilmicosin; PEN, penicillin/AMP, ampicillin/XNL, ceftiofur; CLI, clindamycin; FFN, florfenicol; SXT, trimethoprim sulpha/SDM, sulphadimethoxine; OXY, oxytetracycline; CTET, chlortetracycline; TIA, tiamulin*.

*^a^Categorization of antimicrobial drugs based on importance in human medicine—Veterinary Drug Directorate*.

*^b^Pathogen minimum inhibitory concentration breakpoints from CLSI standards*.

*^c^No CLSI breakpoint for this bovine respiratory disease pathogen/antimicrobial combination, simply notes susceptibility*.

*Values in red indicate resistant proportion of samples*.

*Shaded areas indicate concentrations not tested*.

**Table 4:** Distribution of minimum inhibitory concentrations among *Mycoplasma bovis* isolates (*n* = 211 animals and 226 samples; lung samples = 194, nasal swabs = 23, laryngeal swabs = 1, pleural fluid = 1, heart = 2, joint fluid = 3, peritoneum = 1, abscess = 1).

					Distribution (%) of MICs (µg/mL)									
Class	Category^a^	Antimicrobial	MIC 50	%R^b^	≤0.12	0.25	0.5	1	2	4	8	16	32	64
Aminoglycoside	II	GEN	16	58				1.3	1.3	8	31.4	58		
	II	NEO	32	97.7						1.3	0.9	0.4	97.3	
	III	SPE	8	0.9							88.9	8.8	0.9	64 = 0.4 >64 = 0.9
Fluorquinolone	I	ENRO	0.25	8	44.2	41.6	40	2.2	8					
	I	DANO	0.25	17.7	15.5	42.5	24.3	17.7						
Macrolide	II	TYLT	32	97.7			0.9			0.4	0.9	0.4	97.3	
	II	TUL	64	92				0.4	0.9	0.4	0.9	3.5	1.8	92
	II	TIL	64	98.2						1.3		0.4		98.2
B lactams	I	XNL	8	98.2		1.8					98.2			
	II	PEN	8	98.6	0.13	0.4				0.4	98.2			
	II	AMP	16	98.6		0.9	0.4				0.4	98.2		
Lincosamides	II	CLIN	16	73^c^		8.4	7.1	6.2	4.0	1.3	3.1	69.9		
Phenicol	III	FFN	4	25.7		1.3	1.3	5.8	19.9	46	25.7			
Tetracycline	III	OXY	8	80.1			1.3	0.9	8	9.7	80.1			
	III	CTET	8	69.5			2.2	2.2	7.1	19	69.5			
Pleuromutilin		TIA	1	2.2^c^			42	27.9	11.5	5.3	8.4	2.7	2.2	

*Trimethoprim sulpha (Category II): <2/38 = 2.2%, >2/38 = 97.8%*.

*Sulphahdimethoxine (Category III): <256 = 40.3%, >256 = 59.7%*.

*GEN, gentamycin/NEO, neomycin/SPE, spectinomycin; DAN, danofloxacin/ENRO, enrofloxacin; TYLT, tylosin/TUL, tulathromycin/TIL, tilmicosin; PEN, penicillin/AMP, ampicillin/XNL, ceftiofur; CLI, clindamycin; FFN, florfenicol; SXT, trimethoprim sulpha/SDM, sulphadimethoxine; OXY, oxytetracycline; CTET, chlortetracycline; TIA, tiamulin*.

*^a^Categorization of Antimicrobial Drugs Based on Importance in Human Medicine—Veterinary Drug Directorate*.

*^b^Pathogen minimum inhibitory concentration breakpoints from CLSI standards*.

*^c^No CLSI breakpoint for this bovine respiratory disease pathogen/antimicrobial combination, simply notes susceptibility*.

*Values in red indicate resistant proportion of samples*.

*Shaded areas indicate concentrations not tested*.

**Table 5:** Distribution of minimum inhibitory concentrations among *Pasteurella multocida* isolates (*n* = 113 animals and 117 samples; lung samples = 85, nasal swabs = 30, laryngeal swabs = 1, joint fluid = 1).

					Distribution (%) of MICs (µg/mL)									
Class	Category^a^	Antimicrobial	MIC 50	%R^b^	≤0.12	0.25	0.5	1	2	4	8	16	32	64
Aminoglycoside	II	GEN	4	8.5^c^					8.5	53.0	29.9	8.5		
	II	NEO	16	65.8^c^						5.1	29.1	28.2	37.6	
	III	SPE	32	27.4							1.7	36.8	32.5	64 >64 1.7 27.4
Fluorquinolone	I	ENRO	0.12	0	91.5	6.0	1.7	0.9						
	I	DANO	0.12	1.7^c^	88.9	6.8	2.6	1.7						
Macrolide	II	TYLT	32	99.1^c^							0.9	6.8	92.3	
	II	TUL	4	29.9				13.7	30.8	11.1	5.1	7.7	1.7	29.9
	II	TIL	16	41.9						12.9	33.3	12.0	0.9	41.0
B lactams	I	XNL	0.25	0.9		94.9	0.9	2.6		0.9	0.9			
	II	PEN	0.25	1.7	30.8	56.4	11.1				1.7			
	II	AMP	0.25	1.8^c^		72.6	24.8	0.9	0.9		0.9			
Lincosamides	II	CLIN	16	100^c^							1.7	98.3		
Phenicol	III	FFN	0.5	1.7		10.3	58.1	25.6	4.3		1.7			
Tetracycline	III	OXY	8	55.6			29.1	7.7	3.4	4.3	55.6			
	III	CTET	4	43.6			16.2	21.4	11.1	7.7	43.6			
Pleuromutilin		TIA	32	86.3^c^						0.9	0.9	12.0	86.3	

*Trimethoprim sulpha (Category II): <2/38 = 76.1%, >2/38 = 23.9%*.

*Sulphahdimethoxine (Category III): <256 = 35.9%, >256 = 64.1%*.

*GEN, gentamycin/NEO, neomycin/SPE, spectinomycin; DAN, danofloxacin/ENRO, enrofloxacin; TYLT, tylosin/TUL, tulathromycin/TIL, tilmicosin; PEN, penicillin/AMP, ampicillin/XNL, ceftiofur; CLI, clindamycin; FFN, florfenicol; SXT, trimethoprim sulpha/SDM, sulphadimethoxine; OXY, oxytetracycline; CTET, chlortetracycline; TIA, tiamulin*.

*^a^Categorization of antimicrobial drugs based on importance in human medicine—Veterinary Drug Directorate*.

*^b^Pathogen minimum inhibitory concentration breakpoints from CLSI standards*.

*^c^No CLSI breakpoint for this bovine respiratory disease pathogen/antimicrobial combination, simply notes susceptibility*.

*Values in red indicate resistant proportion of samples*.

*Shaded areas indicate concentrations not tested*.

**Table 6:** Distribution of minimum inhibitory concentrations among *Histophilus somni* isolates (*n* = 72 animals and 75 samples, lung samples = 63, nasal swabs = 5, pleural fluid = 1, heart/pericardium = 5, abscess = 1).

					Distribution (%) of MICs (µg/mL)									
Class	Category^a^	Antimicrobial	MIC 50	%R^b^	≤0.12	0.25	0.5	1	2	4	8	16	32	≥64
Aminoglycoside	II	GEN	8	32.0^c^				8.0	9.3	20.0	30.7	32		
	II	NEO	32	85.3^c^						5.3	9.3	13.3	72.0	
	III	SPE	16	10.7							17.3	48.0	24.0	10.7
Fluorquinolone	I	ENRO	0.12	4.0	88.0	1.3	1.3	5.3	4.0					
	I	DANO	0.12	10.7^c^	84.0	5.3		10.7						
Macrolide	II	TYLT	8	34.6^c^			2.7	5.3	13.3	24.0	20.0	21.3	13.3	
	II	TUL	8	21.3				2.7	10.7	33.3	25.3	6.7		21.3
	II	TIL	4	18.7						54.7	24.0	2.7		18.7
B lactams	I	XNL	0.25	0		97.3	2.7							
	II	PEN	0.12	13.3	84.0	2.7			4.0	4.0	5.3			
	II	AMP	0.25	11.9^c^		85.3		2.7	5.3	1.3	1.3	4.0		
Lincosamides	II	CLIN	1	12.0^c^		13.3	37.3	32.0	4.0	1.3		12.0		
Phenicol	III	FFN	0.25	1.3		78.7	9.3	10.7			1.3			
Tetracycline	III	OXY	8	54.7			20.0	4.0	10.7	10.7	54.7			
	III	CTET	2	16.0			26.7	10.7	22.7	24.0	16.0			
Pleuromutilin		TIA	1	0^c^			16.0	25.3	48.0	8.0	1.3	1.3		

*Trimethoprim sulpha^4^ (Category II): <2/38 = 97.3%, >2/38 = 2.7%*.

*Sulphahdimethoxine^4^ (Category III): <256 = 44.0%, >256 = 56.0%*.

*GEN, gentamycin/NEO, neomycin/SPE, spectinomycin; DAN, danofloxacin/ENRO, enrofloxacin; TYLT, tylosin/TUL, tulathromycin/TIL, tilmicosin; PEN, penicillin/AMP, ampicillin/XNL, ceftiofur; CLI, clindamycin; FFN, florfenicol; SXT, trimethoprim sulpha/SDM, sulphadimethoxine; OXY, oxytetracycline; CTET, chlortetracycline; TIA, tiamulin*.

*^a^Categorization of antimicrobial drugs based on importance in human medicine—Veterinary Drug Directorate*.

*^b^Pathogen minimum inhibitory concentration breakpoints from CLSI standards*.

*^c^No CLSI breakpoint for this bovine respiratory disease pathogen/antimicrobial combination, simply notes susceptibility*.

*Values in red indicate resistant proportion of samples*.

*Shaded areas indicate concentrations not tested*.

**Table 7:** Distribution of minimum inhibitory concentrations among *Trueperella pyogenes* isolates (*n* = 83 animals and 94 samples; lung samples = 69, nasal swabs = 15, laryngeal swab = 2, heart/pericardium = 1, joint fluid = 7).

					Distribution (%) of MICs (µg/mL)									
Class	Category^a^	Antimicrobial	MIC 50	%R^b^	≤0.12	0.25	0.5	1	2	4	8	16	32	≥64
Aminoglycoside	II	GEN	1	9.6				83.0	3.2	2.1	2.1	9.6		
	II	NEO	4	9.6						84.0	6.4	1.1	8.5	
	III	SPE	8	1.1							95.7	2.1	1.1	1.1
Fluorquinolone	I	ENRO	1	0			43.6	54.3						
	I	DANO	1	91.5	3.2		5.3	91.5						
Macrolide	II	TYLT	32	79.7			16.0		1.1		3.2	10.6	69.1	
	II	TUL	64	57.4				23.4	4.3	3.2	5.3	5.3	1.1	57.4
	II	TIL	64	75.6						22.3	2.1		1.1	74.5
B lactams	I	XNL	0.5	1.1		12.8	40.4	42.6	2.1	1.1	1.1			
	II	PEN	0.12	1.1	94.7	2.1	2.1				1.1			
	II	AMP	0.25	1.1		95.8	2.1	1.1				1.1		
Lincosamides	II	CLIN	16	83.0		12.8		2.1	1.1	1.1		83.0		
Phenicol	III	FFN	1	30.9		2.1	25.5	27.7	7.4	6.4	30.9			
Tetracycline	III	OXY	8	94.7			1.1	2.1		2.1	94.7			
	III	CTET	8	92.6			2.1		3.2	2.1	92.6			
Pleuromutilin	III	TIA	0.5	1.1			93.6	1.1		1.1		3.2	1.1	

*Trimethoprim sulpha^4^ (Category II): <2/38 = 98.9%, >2/38 = 1.1%*.

*Sulphahdimethoxine^4^ (Category III): <256 = 18.1%, >256 = 81.9%*.

*GEN, gentamycin/NEO, neomycin/SPE, spectinomycin; DAN, danofloxacin/ENRO, enrofloxacin; TYLT, tylosin/TUL, tulathromycin/TIL, tilmicosin; PEN, penicillin/AMP, ampicillin/XNL, ceftiofur; CLI, clindamycin; FFN, florfenicol; SXT, trimethoprim sulpha/SDM, sulphadimethoxine; OXY, oxytetracycline; CTET, chlortetracycline; TIA, tiamulin*.

*^a^Categorization of antimicrobial drugs based on importance in human medicine—Veterinary Drug Directorate*.

*^b^Pathogen minimum inhibitory concentration breakpoints from CLSI standards*.

*Values in red indicate resistant proportion of samples*.

*Shaded areas indicate concentrations not tested*.

### Analysis

The frequencies of multiclass resistance in the isolates are shown in Table 8. Multidrug resistance was high in all the BRD pathogens with 95.6% resistant to two or more classes and 47.2% resistant to four or five antimicrobial classes. Extreme multidrug resistance occurred in 9.9% of *M*. *haemolytica*, 30.5% of *M*. *bovis*, 41% of *P*. *multocida*, 6.7% of *H*. *somni* and 36.2% of *T*. *pyogenes* isolates. *M. haemolytica* had the highest degree of XDR, with six isolates resistant to eight to nine drug classes. Because of its importance as a therapy for BRD, the frequency of BRD isolates that exhibited multidrug resistance that included tulathromycin resistance are presented in Table 9.

**Table 8:** Number of antimicrobial classes in resistance patterns for important Bovine respiratory disease pathogens.

Isolate	Number of isolates	Number of isolates by number of antimicrobial classes in the resistance pattern					
		0	1	2−3	4−5	6−7	8−9
*Mannheimia haemolytica*	233	1	14	88	107	17	6
*Mycoplasma bovis*	226	0	1	18	138	69	0
*Pasteurella multocida*	117	0	0	24	45	48	0
*Histophilus somni*	75	3	14	33	20	5	0
*Trueperella pyogenes*	94	0	0	18	42	34	0

**Table 9:** Number of isolates resistant to tulathromycin as well as other antimicrobials.

		*Mannheimia haemolytica* (%)	*Mycoplasma bovis* (%)	*Pasteurella multocida* (%)	*Histophilus somni* (%)	*Trueperella pyogenes* (%)
Number of isolates resistant to TUL		88 (37.8)	208 (92)	35 (29.9)	16 (21.3)	54 (57.4)

Number of isolates also resistant to	SPE	9	1	29	2	1
	GEN	8	125	4	11	4
	NEO	78	204	34	16	9
	ENRO	6	17	0	3	0
	DANO	7	39	0	8	50
	TYLT	88	204	35	15	53
	TIL	85	207	35	14	52
	PEN	9	207	35	1	0
	AMP	8	207	0	1	1
	XNL	1	206	0	0	1
	CLI	58	120	35	8	54
	FFN	7	57	2	1	24
	SXT	76	128	33	14	46
	TMS	1	203	21	0	1
	OXY	80	151	32	11	54
	CTET	19	172	18	8	53
	TIA	15	5	34	0	1

We compared the distribution of the MDR for the isolates at Feedlot A and Feedlot B (Table 10). Feedlot B had a significantly (α = 0.05) higher rate of resistance between for *M. haemolytica* (*z* (96) = 3.788, *p* = 0.0002) and *P. multocida* (*z* (57) = -3.183, *p* = 0.0015).

**Table 10:** Comparing the number of antimicrobial classes to which the target organisms were resistant at Feedlot A and Feedlot B using the two sample Wilcoxon rank-sum test.

	Number of isolates of each pathogen by the number antimicrobial classes to which they are resistant																Two sample Wilcoxon rank-sum test
	Feedlot A								Feedlot B								
		
Number of classes	0	1	2	3	4	5	6	7	0	1	2	3	4	5	6	7	*p*-Value
*Mannheimia haemolytica*	0	4	8	3	1	6	0	1	0	0	4	4	24	33	8	0	0.0002
*Mycoplasma bovis*	0	1	2	2	6	21	2	5	0	0	0	8	26	25	22	2	0.810
*Pasteurella multocida*	0	0	0	4	3	9	1	0	0	0	0	2	9	4	24	1	0.0015
*Histophilus somni*	0	3	4	4	1	1	2	1	1	2	7	5	6	2	1	0	0.978
*Trueperella pyogenes*	0	0	0	1	2	7	6	2	0	0	1	4	3	7	8	4	0.675

## Discussion

This study was a successful collaboration of private practice veterinarians, industry, government, and a diagnostic laboratory for monitoring AMR in BRD pathogens from feedlot cattle in southern Alberta and quantified the phenotypic AMR in BRD-affected feedlot cattle.

Most animals in this study were calves that had arrived at the feedlots within the previous 60 days would be considered classical cases of shipping fever. *M. haemolytica* is consistently associated with the acute form of fibrinous pneumonia (16), and it is anticipated that it would be the most frequently isolated bacterium from respiratory tract samples. From lung tissues, *M. bovis* was recovered in almost equal numbers to *M. haemolytica*. Fibrinous pneumonia, characteristic of *M. haemolytica*, was diagnosed in 75% of cases where both these bacteria were isolated*;* bronchopneumonia is classically associated with *M. bovis* infection (17). *P. multocida* was recovered more frequently than *M. bovis* from nasal swab samples. This is likely a reflection of *P. multocida’s* ability to competitively exclude other microflora in complex niches and thus thrive in mixed bacterial communities in the nasopharynx (18). Given that *T. pyogenes* and *H. somni* are more frequently associated with systemic infection (19, 20), it is not surprising that we isolated these bacteria more frequently from lung tissue, heart/pericardium, and joint samples than nasopharyngeal swabs.

Difficulties can arise when comparing microbial prevalence data between BRD studies due to the complex etiology of this illness, inconsistencies in the specific agents targeted among studies, and the effects that differing sampling and lab processing strategies have on recovery rates. For example, comparisons against similar surveillance of BRD mortalities performed by Klima et al. (8) report a 74% recovery of *M. haemolytica* from diseased lung tissue, while samples examined by Fulton et al. (21) showed a 25% recovery rate for *M. haemolytica*, a number more in line with the 33% recovery rate observed here. However, immunohistochemical staining performed by Booker et al. (2) suggested the presence of *M. haemolytica* in >90% of cases of peracute, acute, and subacute forms of pneumonia. The same study found *M. bovis* in 40−50% of acute cases while being far more predominant (90% recovery) in fatal, chronic cases of BRD. Overall, the trends in this dataset reflect what is expected of pneumonia typically exhibited in calves within 60 days of arrival; a larger proportion of *Pasteurellaceae* species and a lesser presence of *H. somni* and *T. pyogenes*. The abundance of *M. bovis* in all body sites examined and its co-isolation with other BRD organisms in 205 of 226 BRD cases in this study has also been reported elsewhere (16).

Sixty-six percent of *H. somni* isolates and all isolates of *M. haemolytica, P. multocida, T. pyogenes*, and *M. bovis* were resistant to at least one of the antimicrobials tested, with the majority of all isolates (90.2%) resistant to at least one of the three macrolide drugs tested. Macrolides are currently used for metaphylaxis in groups of high-risk calves at arrival into feedlots and as direct therapies for BRD cases. The levels of resistance seen to macrolide drugs in this study are of concern given both the importance of this drug class to the beef production system and the importance of macrolide drugs in human medicine.

High levels of tetracycline resistance (>90%) and macrolide resistance (>75%) were observed in *T. pyogenes*. The macrolide, tylosin, is frequently used in the US, and in Canada to a lesser extent, as an in-feed additive for the prevention of liver abscesses that can occur in cattle finished on high grain diets (22). In Canada, tetracycline is used more frequently for this same purpose. The resistance profiles obtained from this study indicate that the use of either macrolides or tetracycline drugs for preventing *Trueperella* may be ineffective.

High levels of resistance (>80%) were also observed against tiamulin (TIA) in the *P. multocida* isolates but not in the other bacterial species examined. Tiamulin is a pleuromutilin drug that is used in-feed for swine production as it can prevent swine dysentery, porcine spirochetosis, and *Pasteurella* associated pneumonia. *P. multocida* resistance to tiamulin has been observed in swine lung samples (23). The TIA resistance observed here combined with a lack of pleuromutilin use in beef production systems raises the question as to the origin of some of the AMR observed in this study.

In North America, there is a trend toward increasing frequencies of MDR in pathogens involved in high mortality BRD cases (6, 8, 24, 25). Recent reports examining the molecular basis for AMR in *Pasteurellaceae* strains from BRD mortalities indicate that large mobile elements, linking arrays of resistance genes together, are present in both American and Canadian fed cattle populations (10, 26). These linked AMR genes can be readily promoted and/or maintained by co-selection using any drug to which the bacteria are resistant. The levels of XDR observed in this study in conjunction with the high prevalence of tulathromycin and oxytetracycline resistance observed indicate that the frequent use of these drugs could be selecting for XDR isolates in feedlots. Further genetic characterization will be required for confirmation but the consequences for therapeutic recommendations are important and an issue that will need to be examined further to determine the most effective means to successfully treat cattle while combating AMR development in the Canadian beef-production system.

Currently, macrolides are the industry’s standard for BRD preventative therapy, but the data here suggests that this may have to change in the future to effectively maintain animal health. However, the need to identify both MDR and understand the consequences for intrinsic resistance in some of the bacteria involved in BRD is important. For example, tulathromycin-resistant isolates were co-resistant to oxytetracycline, chlortetracycline, neomycin, and/or sulphadimethoxine, suggesting that the use of any of these antimicrobials as secondary therapies would likely have resulted in treatment failure. Additionally, the use of ceftiofur for treatment of *M. haemolytica* would be a logical choice given the low levels of ceftiofur resistance observed. However, ceftiofur is a Category I drug and widespread use in cattle could be seen as imprudent. Furthermore, *M. bovis* is intrinsically resistant to ceftiofur and if adopted *en masse*, the use of this drug could potentially open an environmental niche for this species, increasing the incidence of *M. bovis* pneumonia.

Extreme multidrug resistance was identified in 24% and resistance to eight or nine classes in 0.81% of the isolates. These isolates are significant for further genotypic research and may not have been recovered with less extensive sampling strategies. The capacity to detect important changes in AMR patterns within circulating bacterial strains is enhanced with molecular subtyping and identification of integrative conjugative elements within XDR strains. With further research, identifying relatively rare XDR strains circulating in the population may be possible with targeted (risk-based) sampling if the characteristics that influence the probability of an animal carrying XDR strains can be identified. The role of metagenomic sequencing in identifying AMR within the larger animal population is also one that will need to be examined. If used appropriately, some of the newer genomics based technologies might be able to help overcome some of the burden associated with microbiological surveillance methods.

It is important to note that, in this study, the cohorts were diseased animals and may not be representative of the broader cattle population that includes healthy and diseased animals. This study selected animals suspected of having BRD or at postmortem and many would have received multiple antimicrobials. Susceptible strains of the target organisms would have been removed from the sample resulting in a higher proportion of resistant isolates. Klima et al. (8) also reported the proportions of pathogens and AMR-pathogens in BRD-affected animals in Alberta, but this study was limited to mortalities. As with most surveillance studies, using the number of isolates recovered as the denominator (proportion) rather than the source population (rate) can bias the results if the isolates vary with the animal population sampled. However, feedlots represent dynamic populations and determining the appropriate population for the denominator may not be possible. Differences such as the timing and use of vaccinations, AMU at the cow-calf level, pre-weaning, ranch-direct or sales yard source, and distance traveled, make identifying “like” populations difficult. Collecting more epidemiological data for each isolate undergoing AMR testing would help to examine and manage these biases (27).

Antimicrobial resistance patterns varied between Feedlot A and Feedlot B. Feedlot A’s veterinarian did not provide AMU data, so it was not possible to characterize the different proportions of AMR seen at each feedlot in terms of AMU. Additional information about how and where the cattle were sourced and different animal management practices could also be useful for understanding variable AMR patterns. This finding highlights the challenge of identifying a representative sample from a region and the complexity of AMR surveillance. Questions remain about the appropriate sample size for AMR studies. AMR has been shown to cluster, influenced by the ecology of the location (28). Therefore, some authors advocate small numbers of samples from many farms to monitor AMR (29). Our results demonstrated heterogeneity of the resistance patterns within, as well as between feedlots, suggesting that generating a representative sample necessitates the sampling of a considerable number of feedlots and animals within feedlot. Bootstrapping methods for examining the influence of different variance estimates and constructing confidence intervals around an estimated prevalence at both the group-level and individual level have been used to determine sufficient sample sizes for AMR studies (30).

Substantial effort and cost is required to collect samples and the associated epidemiological data as well as complete microbiological testing. To promote the optimum use of public resources, AMR surveillance systems must balance benefits with costs and consider alternative designs to generate the most meaningful data and meet the purpose of the system.

Further studies are required to investigate the epidemiological factors that contributed to AMR. Prospective cohort studies that can accurately measure AMU in the individual, its source herd, and other epidemiological factors commonly associated with BRD, could provide measures of associations between exposures and AMR. Notably, there have been relatively few observational studies or randomized trials comparing interventions to manage AMR in veterinary medicine (31–33).

As was seen here, it can be difficult to motivate veterinary practices to provide AMU data. This is an issue that may need to be resolved in the future before real progress can be made toward AMR intervention in the agricultural setting as collection of AMR data in isolation of AMU is of little value in terms of developing strategies that help control the spread of resistance (34). Continued collaboration within and between agriculture and public sectors is necessary for AMU and AMR surveillance, collection of animal health and management information, and data to determine the success of interventions.

Antimicrobial resistance surveillance in beef production can be challenging, specifically when trying to encompass both animal and regional variability. In addition, obtaining both animal metadata and treatment histories from private veterinary practices can be difficult with constraints on veterinary practitioners’ time and reluctance to share information. This study successfully provides an estimate of the current magnitude of AMR in BRD-affected feedlot cattle in Alberta, encompassing samples from a wide geographic range that are representative of different veterinary practices from this region. The results demonstrated the challenge of effective antimicrobial management in these animals.

## Ethics Statement

This study was exempt from Animal Care Review. This was an observational, cross-sectional study. The animals that were sampled for the study were ill or had died of naturally occurring BRD. Most of the animals examined were mortalities. Sample collection from morbid animals was considered within the normal scope of practice.

## Author Contributions

RMA made significant contributions to the design of the project, was responsible for the analysis and interpretation of the work and drafting and revising the manuscript including the final version to be published. She agrees to be accountable for all aspects of the work and will ensure that questions related to accuracy are investigated and resolved. CK contributed to the interpretation of data, and drafting and revising the manuscript including the final version. She agrees to be accountable for all aspects of the work and will ensure that questions related to accuracy are investigated and resolved. NA, HM-B, CS, and PA contributed to the concept and design of the study, were responsible for data acquisition and participated in critical review of the manuscript including the final version. They will be accountable for all aspects of the work and will ensure that questions related to accuracy are investigated and resolved. SO, DP, KS, MO, TM, and BR contributed to the concept and design of the study, interpretation of the data, and critical review of the manuscript including the final version. They will be accountable for the work and will ensure that questions related to accuracy are investigated and resolved.

## Conflict of Interest Statement

RMA is the owner and director of POV Inc., a professional corporation. She was hired as a consultant, funded by the Growing Forward2 Grant, to provide expertise in the study design, analysis, and interpretation of the data, writing the report and decision to submit the report for publication. POV and MR did not receive payment from a third party for any aspect of the submitted work. She had full access to the data throughout the study and takes responsibility for the integrity of the data and the accuracy of the data analysis. NA and MO are owners and directors of Chinook Contract Research (CCR). CCR administered the Growing Forward2 grant that covered the cost of HM-B to work as a laboratory technician on the project. CCR contributed all overhead costs associated with maintenance of the grant and human resources. CCR and its directors did not receive payment from a third party for any aspect of the submitted work. The directors and HM-B had full access to the data throughout the study and take responsibility for the integrity of the data and accuracy of the data analysis. Institute of Applied Poultry Technologies (IAPT) is a not-for-profit organization of poultry industry stakeholders. At the time of the sample collection and analysis for this project, NA and MO were directors of IAPT. IAPT administered the Alberta Livestock and Meat Agency part of the grant to cover the materials costs of the project and the cost of PA to work as a laboratory technician on the project. Labor and overhead were covered as direct contributions by IAPT. IAPT and its directors did not receive payment from a third party for any aspect of the submitted work. The directors and PA had full access to the data throughout the study and take responsibility for the integrity of the data and accuracy of the data analysis. Bow Valley Research (BVR) is owned by MO. BVR was provided funds through the Growing Forward2 grant that covered the costs of Crystal Schatz to work as a laboratory technician on the project. BVR also donated technical expertise and equipment to the project. BVR and its owner did not receive payment from a third party for any aspect of the submitted work. The directors and CS had full access to the data throughout the study and take responsibility for the integrity of the data and accuracy of the data analysis. MO is owner and director of Alberta Veterinary Laboratories (AVL). AVL donated technical expertise and equipment to the project. AVL and its director did not receive payment from a third party for any aspect of the submitted work. MO had full access to the data throughout the study and take responsibility for the integrity of the data and accuracy of the data analysis. All other authors declares that the research was conducted in the absence of any commercial or financial relationships that could be construed as a potential conflict of interest.
